# A case of immunotactoid glomerulopathy with false-negative IgG staining

**DOI:** 10.1186/s12882-018-0931-4

**Published:** 2018-06-15

**Authors:** Shuma Hirashio, Takahiro Arima, Ayaka Satoh, Kouichi Mandai, Shigeo Hara, Takao Masaki

**Affiliations:** 1 Department of Nephrology, Hiroshima University Hospital, 1-2-3 Kasumi, Minami-ku, Hiroshima, 7348551 Japan; 2 Department of Nephrology, National Hospital Organization Higashihiroshima Medical Center, Hiroshima, Japan; 3 Department of Diagnostic Pathology, National Hospital Organization Higashihiroshima Medical Center, Hiroshima, Japan; 4 Department of Diagnostic Pathology, Kobe University Graduate School of Medicine, Kobe, Japan

**Keywords:** Immunotactoid glomerulopathy, False-negative, Monoclonal gammopathy of undetermined significance, Monoclonal gammopathy of renal significance

## Abstract

**Background:**

Immunotactoid glomerulopathy (ITG) is a rare glomerulonephritis characterized by microtubular deposits. Immunofluorescence findings are necessary to differentiate ITG from other proliferative glomerular diseases. The characteristic tubular structure on electron microscopy is essential for a definitive diagnosis, and the diameter of the structure has been traditionally used for differentiating between ITG and other types of glomerulonephritis with organized deposits. In recent years, the disease concept of monoclonal gammopathy of renal significance, which is associated with M protein produced by plasma cell tumors, has been proposed.

**Case presentation:**

This was a peculiar case of ITG with underlying monoclonal gammopathy in which IgG showed a false-negative result with immunofluorescence using frozen sections. Additional examinations using a different clone of the anti-IgG antibody revealed typical IgG staining. C4d was strongly positive, consistent with immune complex type glomerulonephritis.

**Conclusions:**

This case highlights unusual features of ITG, and provides a practical hint to avoid a diagnostic pitfall.

## Background

Immunotactoid glomerulopathy (ITG) is a rare glomerulonephritis characterized by the appearance of a membranoproliferative pattern, negative amyloid staining, and a characteristic ultrastructure on electron microscopy [1]. ITG was initially considered the same as fibrillary glomerulonephritis, a type of glomerulonephritis with organized deposits comprising randomly disposed fibril substructures [2]. Recent studies suggest that ITG and fibrillary glomerulonephritis are two distinct entities [3–5]. Deposits in fibrillary glomerulonephritis lack the microtubular structure, which is a fundamental feature of ITG, and the diameter of the organized deposits in ITG tend to be greater than that in fibrillary glomerulonephritis [6]. With light microscopy, ITG mainly exhibits a membranoproliferative-like injury pattern, and specific diseases enter the differential diagnosis; for example, cryoglobulinemic glomerulonephritis, light-chain deposition disease, fibronectin glomerulopathy, and C3 glomerulopathy. To differentiate proliferative glomerulonephritis from other disease entities, diagnosis of ITG requires combined immunofluorescence studies and electron microscopy [7, 8]. In the diagnosis of glomerulonephritis, immunofluorescence studies of frozen sections provide critical information on the components of the immune complexes. In ITG, IgG, C3, and, less frequently, C1q, are positive in the mesangium and capillary walls [8, 9].

Here we describe a peculiar case of ITG that showed C3 and C1q staining on initial studies. Subsequent studies, however, revealed glomerular IgG positivity using a different clone of the anti-IgG antibody. This case highlights a diagnostic pitfall in the interpretation of immunofluorescence studies, especially in the diagnosis of C3 glomerulopathy.

## Case presentation

The patient was a 69-year-old man with no history of urinary abnormalities or renal dysfunction. When he was 68, he underwent his first health checkup in several years and was found to have occult blood in his urine, proteinuria, and renal dysfunction. Urinalysis at the first examination showed urine protein of 0.49 g/gCr, urine red blood cells of 30–49/high-power field, and pathological granular casts, for which we decided to perform further studies including a renal biopsy. The patient had a history of untreated dyslipidemia. His family history was unremarkable. The patient was not taking any regular medication at the time of the first examination. Physical findings at the first examination were unremarkable. His blood pressure was normal (112/66 mmHg). There was no edema, lymph node involvement, splenomegaly, purpura, or bone pain. Table 1 shows the results of urinary and blood analyses on admission for the purposes of the renal biopsy (dipstick test for occult blood 2+, urine protein 2+, and urine protein-to-creatinine ratio 0.30 g/g on a spot measurement). The number of dysmorphic red blood cells was 20–29 per high-power field. There were no abnormalities in complete blood count or the blood coagulation system. Serum urea nitrogen was 14.9 mg/dL, serum creatinine was 1.19 mg/dL, and estimated glomerular filtration rate by creatinine was 47.6 mL/min/1.73 m^2^. Serum cystatin C level was 1.73 mg/L and estimated glomerular filtration rate by cystatin was 37.1 mL/min/1.73 m^2^. Immunoglobulin levels were normal. Autoantibodies were negative. Serum and urine monoclonal immunoglobulin (immunofixation electrophoresis) were positive. The serum levels of the IgG κ and λ chains were 31.40 mg/dL and 33.60 mg/dL, respectively. The κ/λ ratio was 0.935. Serum cryoglobulin was negative.

**Table 1:** Laboratory results on admission

Parameter	Value	Reference range
Urine		
Red blood cell (/HPF)	50–99	< 5
Urine protein/creatinine ratio (g/g)	0.32	< 0.15
Urine protein electrophoresis	Positive (κ & λ)	Negative
Blood		
Leukocyte count (/μL)	7600	4500–9000
Hemoglobin (g/dL)	13.0	13.6–17.0
Platelet count (× 10^4^/μL)	32.1	14–36
Urea nitrogen (mg/dL)	14.9	8.0–22.0
Creatinine (mg/dL)	1.19	0.60–1.10
Uric acid (mg/dL)	6.8	3.6–7.0
Total protein (g/dL)	7.2	6.7–8.3
Albumin (g/dL)	4.1	4.0–5.0
Lactate dehydrogenase (IU/L)	162	119–229
Sodium (mEq/L)	140	138–146
Potassium (mEq/L)	4.8	3.6–4.9
Chloride (mEq/L)	104	99–109
Corrected serum calcium (mg/dL)	9.3	8.6–10.4
Phosphate (mg/dL)	3.2	2.5–4.7
C-reactive protein (mg/dL)	0.06	< 0.30
IgG (mg/dL)	1045	870–1700
IgA (mg/dL)	317	110–410
IgM (mg/dL)	444	33–190
IgE (IU/mL)	131.9	< 269
light-chain type κ (mg/L)	31.40	2.42–18.92
type λ (mg/L)	33.60	4.44–26.18
Serum protein electrophoresis	Positive (κ & λ)	Negative
CH50 (CH50/mL)	41.5	25–48
C3 (mg/dL)	115	65–135
C4 (mg/dL)	35	13–35
IC (C1q) (μg/mL)	< 1.5	< 1.5
Cryoglobulin	Negative	Negative
Anti-nuclear antibody	< 80×	< 80×
Anti-ds DNA antibody (IU/mL)	2.5	< 10.0
PR3-ANCA (U/mL)	< 1.0	< 3.5
MPO-ANCA (U/mL)	< 1.0	< 3.5
Anti–GBM antibody (U/ml)	<7.0	<7.0
Tissue of bone marrow	Normal tissue component	

*HPF* high-power field, *WF* whole field, *IgG* immunoglobulin G, *IgA* immunoglobulin A, *IgM* immunoglobulin M, *IgE* immunoglobulin E, *C1q-IC* C1q binding IgG immune complex, *ds* double stranded, *PR3* proteinase 3, *MPO* myeloperoxidase, *ANCA* antineutrophil cytoplasmic antibodies, *GBM* glomerular basement membrane

**Fig. 1:**
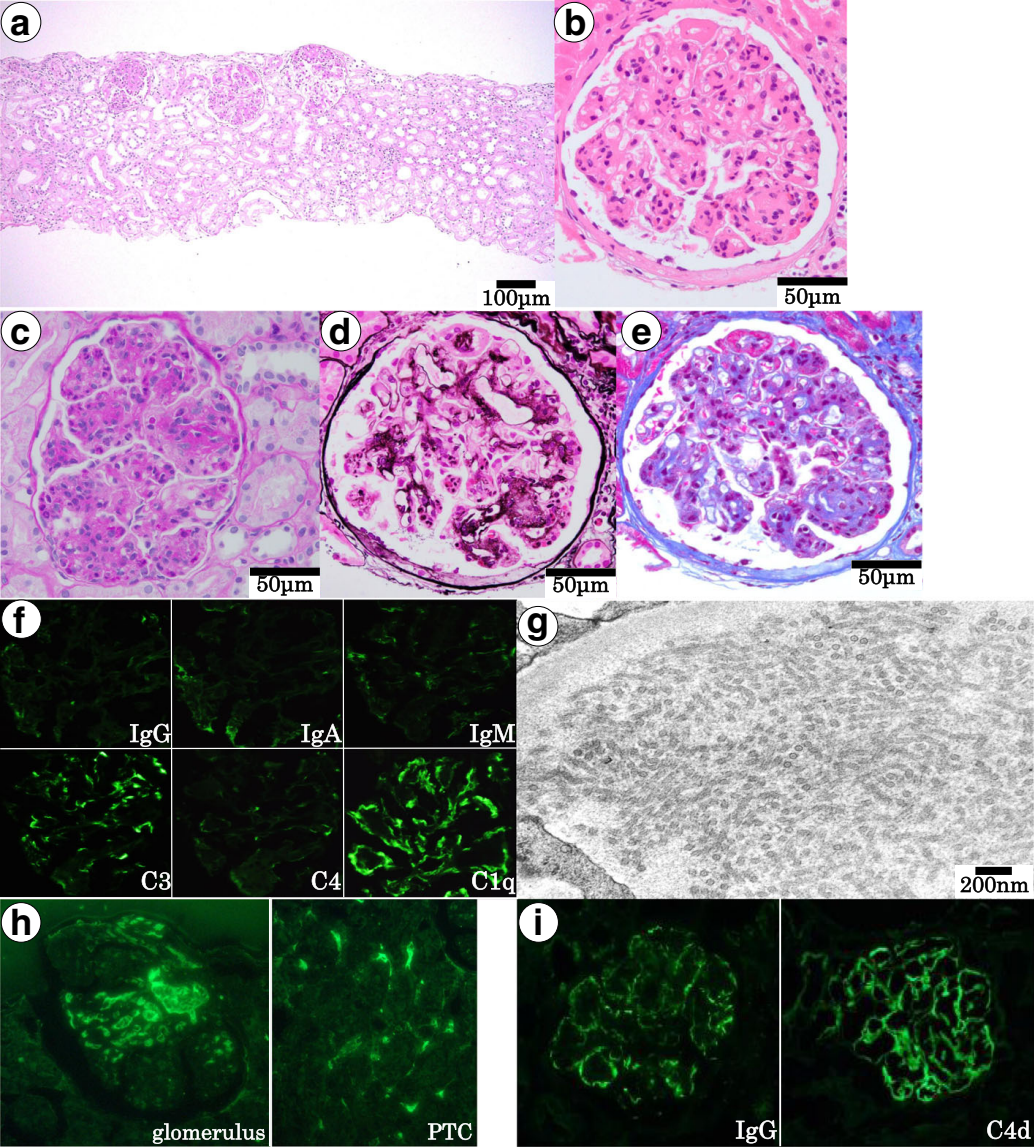
Diagnostic studies. (**a**) Periodic acid–Schiff (PAS) staining at a low magnification (× 100). All glomeruli exhibited lobular accentuation. (**b–e**) Glomerular images of (**b**) hematoxylin-eosin, (**c**) PAS, (**d**) Periodic acid–methenamine-silver, and (**e**) Masson’s trichrome staining (×400). Glomeruli show endocapillary hypercellularity with neutrophilic and eosinophilic infiltration. Scale bar: 100 μm (**a**) and 50 μm (**b–e**). (**f**) Immunofluorescence studies using frozen sections. Immunoglobulins were negative. C3 (1+) and C1q (2+) were positive on glomerular capillaries. (**g**) Electron microscopy image. Cross-sectional view of deposits with microtubular structures. The distribution of these structures was segmental. Scale bar: 200 nm. (**h**) IgG detection by paraffin-immunofluorescence following pronase pretreatment. The left panel is a glomerulus, and the right panel is a peritubular capillary. The positive signal in the microcapillary lumen was considered to be nonspecific staining for plasma components. (**i**) Immunofluorescence studies using anti-IgG (left panel, 55,144, MP Biomedicals) and anti-C4d (right panel) antibodies. IgG and C4d were positive on glomerular capillaries, corresponding to staining patterns for C3 and C1q

With light microscopy, 54 glomeruli were observed, and two glomeruli showed global sclerosis. The remaining glomeruli were enlarged and showed lobular accentuation. In addition to mesangial cell proliferation, all glomeruli showed prominent endocapillary hypercellularity (Fig. 1a–e). Neutrophils and eosinophils showed marked infiltration. There was no hyaline thrombus, crescent formation, and double contour of the capillary walls. Interstitial fibrosis and tubular atrophy was mild. Direct fast scarlet staining was negative. Immunofluorescence was positive for C3 (1+) and C1q (2+) along the glomerular capillaries. However immunoglobulin G (IgG) was negative. The clone we used to originally test for IgG was produced by Medical & Biological Laboratories Co., Ltd. (Lot No. 104AG; Aich, Japan). All immunoglobulins were negative (Fig. 1f). Electron microscopy revealed marked endocapillary hypercellularity. There was infiltration of polymorphonuclear leukocytes and monocytes, occluding glomerular capillary lumens. In the subendothelial space, there were many tubular structures in parallel arrays. With higher magnification, the microtubules had a hollow core and the diameter was approximately 40 nm (Fig. 1g). Most of the deposits contained microtubular structures.

Based on the characteristic electron microscopy findings, ITG was suspected; however, negative staining for immunoglobulins was an unusual finding for ITG. IgG detection by paraffin-immunofluorescence following pronase pretreatment was strongly positive within the capillary spaces of a glomerulus (Fig. 1h). Immunofluorescence examination of frozen sections using a different clone of anti-IgG antibody (55,144; MP Biomedicals, Tokyo, Japan) showed positive staining in capillary walls. Immunofluorescence for C4d was positive along glomerular capillaries (Fig. 1i). Collectively, the patient was diagnosed with ITG with false-negative IgG staining.

Additional laboratory data revealed that serum and urine were positive for monoclonal immunoglobulins. In a bone marrow biopsy specimen, the proportion of plasma cells was 1.6%, excluding plasma cell myeloma. Chromosomal aberrations were not found. 18F-fluoro-deoxy-glucose positron emission tomography demonstrated no significant uptake.

## Discussion and conclusions

ITG is a primary glomerular disease first reported in 1977 by Rosenmann and Eliakim [10]. It is prevalent in Caucasians, accounting for nearly 0.1% of renal biopsy cases. ITG is considered less prevalent in Japan. In general, patients with ITG are likely to have progressive renal dysfunction, and approximately 50% have nephrosis and/or hypertension [6]. Treatment for this condition is generally considered ineffective, although a study reported an amenable effect with steroid treatment in some patients [11].

Electron microscopy is the gold standard for the diagnosis of ITG. Ultrastructural findings in the current case were consistent with ITG (presence of typical tubular structures with a small hollow core inside on short-axis view) [8, 9]. Negative IgG staining is an unusual finding in ITG [8, 12], although some reports have described negative glomerular IgG staining in otherwise typical ITG [13, 14]. Given the possibility of a false-negative result in the initial study using the polyclonal anti-IgG antibody, we conducted IgG staining using a different anti-IgG clone, which revealed positive staining in glomeruli. In addition, C4d was positive in glomeruli, consistent with the features in immune complex type glomerulonephritis [15]. Masked immune deposits in membranous-like nephropathy and membranoproliferative glomerulonephritis have been recently reported [16, 17], indicating the potential diagnostic pitfalls in immunofluorescence studies. In the current case, IgG staining of paraffin-immunofluorescence following pronase pretreatment could not be evaluated because of nonspecific staining of residual serum within the glomerular capillaries [18]. In our case, the successful immunofluorescence study with the application of a different antibody clone probably reflects recognition of the target epitope by the different antibody clone, not “masked antigens”. In addition to pronase digestion, application of different antibody clones to frozen sections may help avoid false negative result in immunofluorescence studies.

A recent study highlights the pathogenetic association between ITG and monoclonal gammopathy due to multiple myeloma and related diseases [19]. Among the pathological conditions regarded as monoclonal gammopathy of undetermined significance without organ dysfunction, monoclonal gammopathy of renal significance has been proposed as a term to refer to a concept of disease manifesting with renal dysfunction during the disease course [20]. Despite the poor proliferative capacity of abnormal plasma cells, monoclonal gammopathy of renal significance is presumably associated with the presence of “dangerous small B-cell clones,” which produce the M protein that is highly likely to be deposited in tissue [21]. The importance of early therapeutic interventions for underlying diseases has been highlighted [19]. The current case represents one manifestation of monoclonal gammopathy of renal significance classified as ITG, and highlights the potential diagnostic pitfall with immunofluorescence studies.

In conclusion, we experienced an unusual case of ITG with false-negative IgG deposits. A definitive diagnosis of ITG was made based on electron microscopy findings, and immunofluorescence results were considered a false-negative result for IgG. In addition to pronase digestion in paraffin sections [15, 16], application of a different antibody clone, as presented in this report, may be another salvage technique in immunofluorescence studies.

## Abbreviation

ITG: Immunotactoid glomerulopathy
